# Supplementary material for: Collapse of the standard ferromagnetic domain structure in hybrid Co/Molecule bilayers
Source: Nat Commun. 2025 Jul 1;16:5807. doi: 10.1038/s41467-025-61068-7 (PMC12218293; doi:10.1038/s41467-025-61068-7)
Supplement: Supplementary file 1 — Supplementary information [file 41467_2025_61068_MOESM1_ESM.pdf]

Supplementary information for: Collapse of the standard ferromagnetic domain structure in hybrid Co/Molecule bilayers

Mattia Benini[1,2] *, Andrei Shumilin[3,4] *, Viktor Kabanov[3], Rajib Kumar Rakshit[1], Antarjami Sahoo[1], Anita Halder[5,6], Andrea Droghetti[5,7], Francesco Cugini[8], Massimo Solzi[8], Diego Bisero[9], Patrizio Graziosi[1], Alberto Riminucci[1], Ilaria Bergenti[1], Manju Singh[1], Luca Gnoli[1], Samuele Sanna[10], Mirko Cinchetti[2], Tomaz Mertelj[3], Stefano Sanvito[5] *, Valentin Alek Dediu[1] *

[1] Istituto per lo Studio dei Materiali Nanostrutturati - CNR (ISMN-CNR), Via Piero Gobetti 101, Bologna 40129, Italy
[2] Department of Physics, TU Dortmund University, Otto-Hahn-Straße 4, 44227 Dortmund, Germany.
[3] Department of Complex Matter, Jozef Stefan Institute, Jamova 39, 1000 Ljubljana, Slovenia
[4] Instituto de Ciencia Molecular, Universitat de València, 46980 Paterna, Spain
[5] School of Physics, AMBER and CRANN Institute, Trinity College, Dublin 2, Ireland
[6] Department of Physics, SRM University-AP, Amaravati, Andhra Pradesh 522240, India
[7] Department of Molecular Sciences and Nanosystems, Ca' Foscari University of Venice, via Torino 155, 30170, Venice-Mestre, Italy
[8] Dept. Mathematical, Physical and Computer Sciences, University of Parma, Parco Area delle Scienze 7/A, 43124 Parma, Italy
[9] Department of Physics and Earth Science, University of Ferrara, Via Saragat 1, I-44122 Ferrara, Italy
[10] Department of Physics and Astronomy "A. Righi", University of Bologna, via Berti-Pichat 6/2, 40126 Bologna, Italy

S.I AMR DATA

Fig. S1 shows the AMR values measured at 80K for the Co(5nm)/Al(4nm) and for Co(5nm)/Gaq_3(25nm) in both transverse (**H** in plane and perpendicular to **I**) and longitudinal (**H** in plane and parallel to **I**), with an applied voltage of 100 mV. The coercivity is extracted from the data by locating the H value corresponding to the AMR peaks. The Co/Gaq_3 sample has a value of (130 ± 1) mT while a value of (5 ± 1) mT is found for the reference Co/Al, in agreement with MOKE measurements performed at the same temperature.

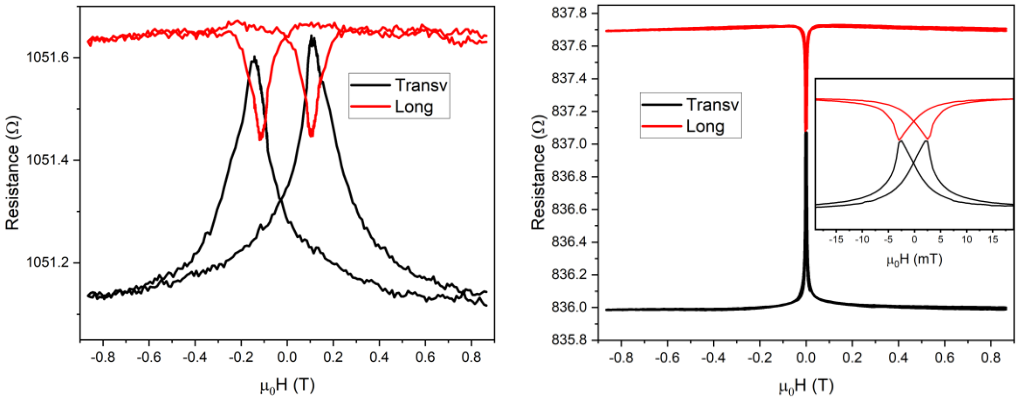

Fig. S1 AMR data for Co/Gaq_3 sample (left) and reference Co/Al (right) obtained at 80 K in both transverse (black lines) and longitudinal (red lines).

S.II MINOR LOOPS CHARACTERIZATION

The minor loops obtained by MOKE magnetometry at 150K are reported in **Fig. S2**, while the ones obtained by SQUID magnetometry are reported in **Fig. S3**. Note that the loops clearly indicate the absence of any detectable second magnetic phase. The non-normalized plot of coercive field vs maximum applied field (data used for **Fig. 2b** in the man article) is displayed in **Fig. S4**, with added data for Co/CoOx for completeness. It is worth noting that the values extracted from Co/Gaq_3 and Co/C_{60} by SQUID measurements are lower in absolute value with respect to the ones obtained by MOKE measurements. This mismatch is attributed to SQUID measurements not being done with the applied field along the samples easy axis, thus resulting in a lowering of the measured coercivity.

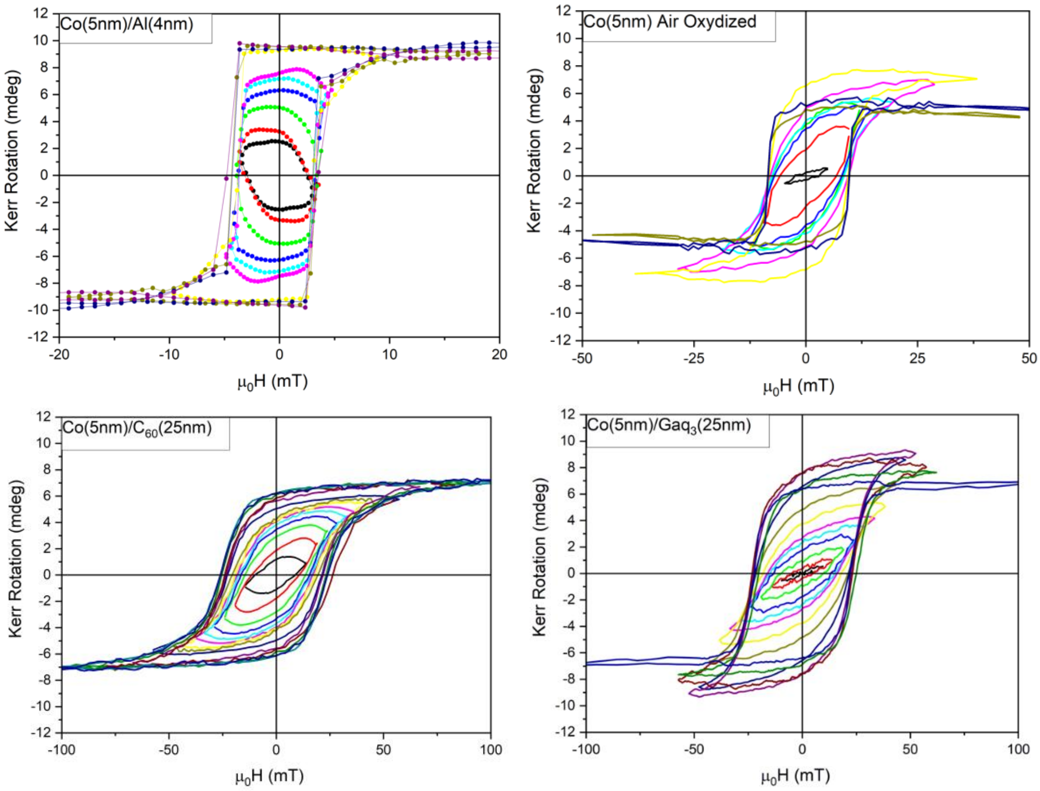

Fig. S2 Minor loops at 150K for reference Co/Al (top-left) Co/CoOx (top-right). Co/C_{60} (bottom-left) and Co/Gaq_3 (bottom-right). The x-scale is varied for each sample for better evidence of the smaller loops.

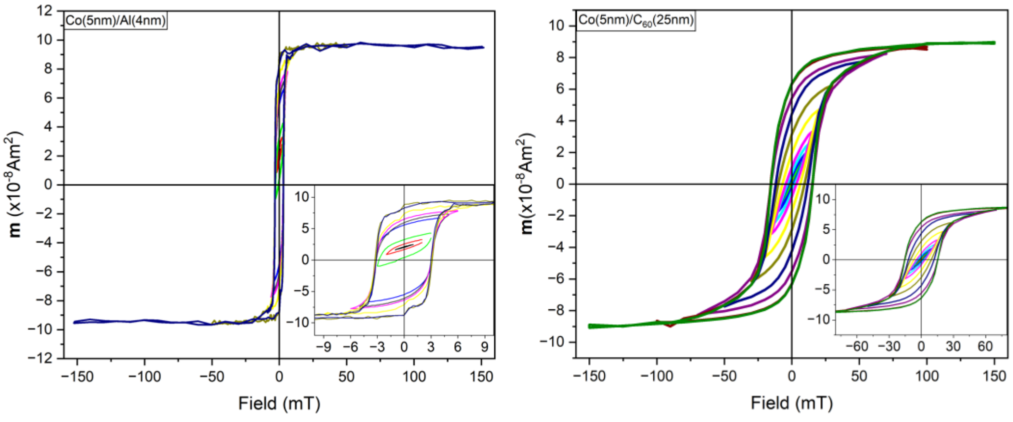

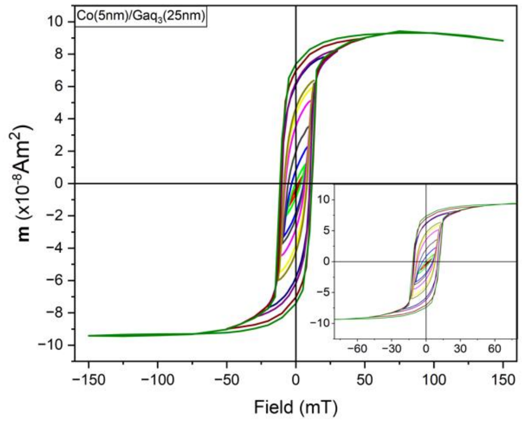

Fig. S3 Minor loops for the Co/C_{60}, Co/Gaq_3 and reference Co/Al obtained by SQUID magnetometry at T=150K. Insets show the zoomed-in central part of the graphs for better evidence of the smaller loops (quantities and units of x and y axis are the corresponding of the non-zoomed graph).

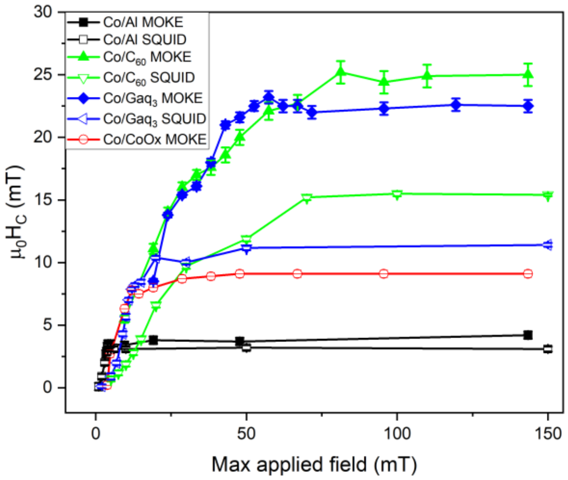

Fig. S4 Absolute values of the coercivity as a function of the minimum applied field in each loop. Error bars indicate the resolution of the magnetic field sweep.

S.III DFT CALCULATIONS, CALCULATED ATOM RESOLVED ANISOTROPY

We demonstrate here that the enhancement of E_{MCA} predicted by DFT in Co/Gaq_3 is an interface effect induced by the molecule. We resolve the contribution of each atom *i* to E_{MCA} by computing the difference of the SOC energy per atom, $E_{MCA}(i) \propto \Delta E_{SOC}(i) = E_{SOC}(i, \theta_{EASY}) - E_{SOC}(i, \theta_{HARD})$. Negative (positive) values of $\Delta E_{SOC}(i)$ indicate contributions that increase (reduce) the slab's MCA. As shown in **Fig. S5**, $\Delta E_{SOC}(i)$ fluctuates. Nevertheless, it has the largest value for a few surface atoms, which are mostly those forming a covalent bond with the molecule. Hence, these atoms contribute the most to the MCA. This result can be highlighted by carrying out a partial summation $S(N') = \sum_{i=1}^{N'} \Delta E_{SOC}(i)$ with $N' \leq N$. $S(N')$ is rather small when considering only the atoms in the bottom layers of the slab, while it jumps of an order of magnitude when reaching interface layer.

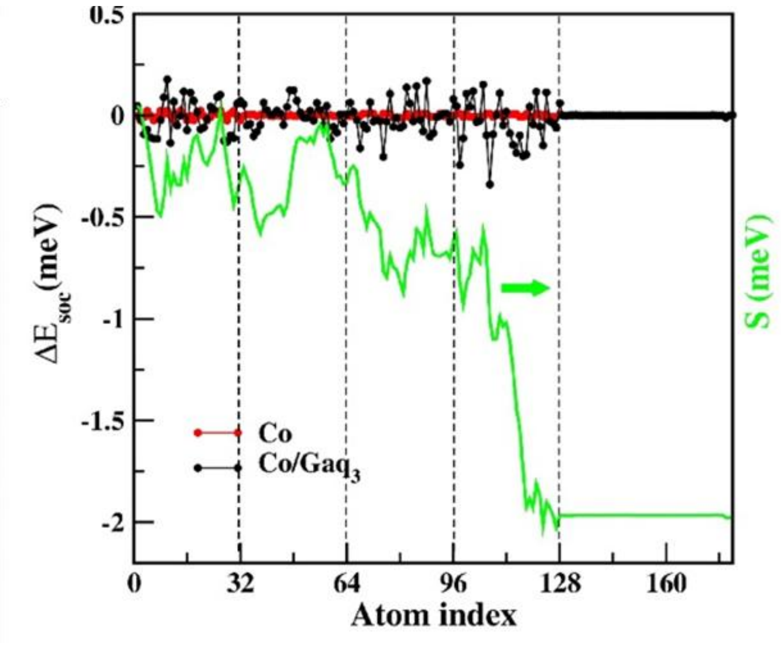

Fig S.5 SOC energy difference $\Delta E_{SOC}(i) = E_{SOC}(i, \theta_{EASY}) - E_{SOC}(i, \theta_{HARD})$ per atom i=1, …,180. Red and Black points are respectively for the clean Co slab and the Co/Gaq$_3$ slab. Atoms labelled from 96 to 128 are at top surface layer, where the molecule is adsorbed, whereas atoms from 0 to 32 are at the bottom surface. Atoms labelled from 128 to 180 are the atoms of the molecule. The green curve is the cumulative sum $S(N') = \sum_{i=1}^{N'} \Delta E_{SOC}(i)$ used to highlight that the largest contribution to the MCA comes from the interface atoms.

S.IV MACROSCOPIC-SCALE CALCULATIONS

To study the model described by Eq. (1) in the main text, we introduce the discrete lattice that can be considered as its mapping to the "classical spin glass" model[1,2]. It allows us to use a variety of conventional numerical methods for its study. However, the mapping implies that the anisotropies on neighbour sites are strongly correlated. The step of the lattice should be small compared to Δ, while the size of the whole lattice should be large compared to the features of magnetic state. It limits the values of r_c that are possible to simulate with our methods. All the results present in the article correspond to 128x128 lattice with size L=60Δ. They are checked to be robust against modification of lattice size and step.

The calculations start with the generation of random but correlated anisotropy field that was achieved with NAG library. First, we generate two random fields a_x and a_y with the Gaussian variogram using the NAG routine g05zrf with correlation length r_c. The distribution of the random angle ϕ was calculated as:

$$\phi = \arctan(\frac{a_y}{a_x}). \quad (S1)$$

As a result, the random field ϕ is correlated with the radius r_c. A typical distribution of the anisotropy angles is presented in **Fig. S6 (b)**. The correlation function of the anisotropy angles is plotted in **Fig. S6(a)**. It is clear that the spatial distribution of the anisotropy angles is not Gaussian but the correlation function is decreasing on the scale of r_c.

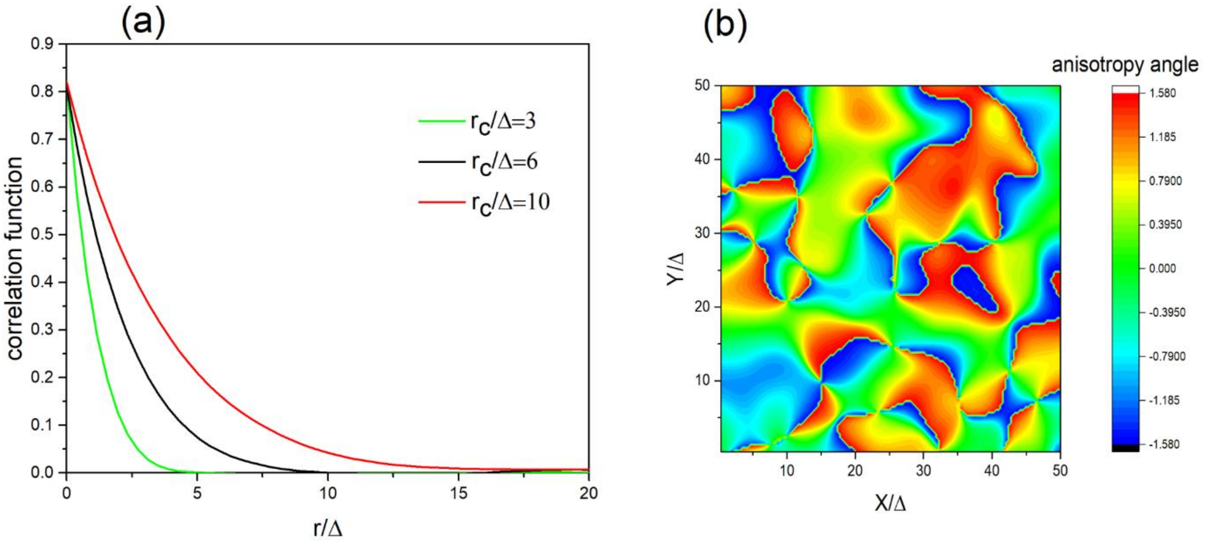

Fig. S6 (a) Dependence of the angle correlation function on distance. **(b)** Distribution of the anisotropy field over the sample for $r_C/\Delta = 4$.

The magnetic states are found by the minimization of the free energy integrated over the sample surface.

$$\int \mathcal{F} d^3 r \to min \qquad (S2)$$

that is done numerically with the conjugate gradient method.

The ferromagnetic material described with Eq. (1) in the main text has many local minima of free energy at small magnetic fields. Therefore, the local minimum found with the numeric procedure depends on the initial state of the system. When the external field $\mathbf{H}$ is changed adiabatically by the small value ($\mathbf{H} \to \mathbf{H} + \delta\mathbf{H}$) the minimum for previous field $\mathbf{H}$ can be used as an initial state to the modified field $\mathbf{H} + \delta\mathbf{H}$. For the calculation of the full hysteresis loops the procedure starts from a high magnetic field $H \gtrsim K_R M_0$ and the uniform saturated magnetic state, $\mathbf{M} = M_0 \mathbf{e}_x$.

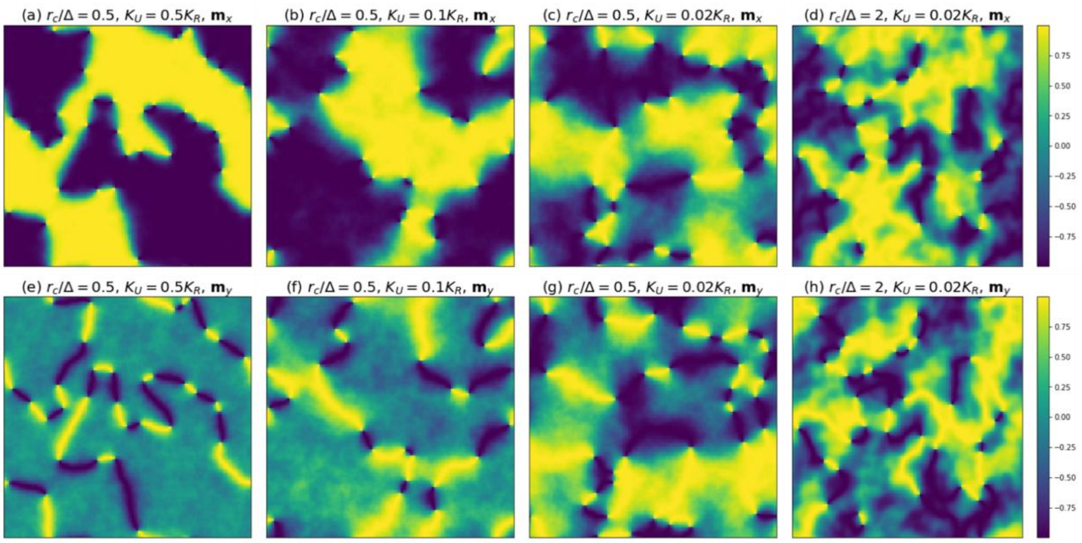

Fig. S7 Demagnetized states for different parameters r_c/Δ and K_U/K_R.

However, the calculations of minor loops should start with a demagnetized state of the film that is not known *a priori*. To find it we apply a "numerical cooling" procedure to our system. It is the solution of Landau-Lifshitz-Gilbert (LLG) equation with additional noise.

$$\frac{\mathrm{d}\varphi(\mathbf{r})}{\mathrm{d}t} = -\gamma\lambda|\tilde{\mathbf{h}}|\sin(\varphi(\mathbf{r}) - \varphi_h) + \zeta(\mathbf{r}, t) \qquad \text{(S3)}$$

Here $\varphi(\mathbf{r})$ is the polar angle describing the direction of magnetization. $\tilde{\boldsymbol{h}}$ is the dimensionless effective field $\tilde{\mathbf{h}} = (\partial\mathcal{F}/\partial\mathbf{M})/M_0$ and φ_h is its polar angle. γ is the gyromagnetic ratio and λ is damping parameter. $\zeta(\boldsymbol{r}, t)$ is the random force with the correlation function

$$\langle\zeta(\mathbf{r}, t)\zeta(\mathbf{r}', t')\rangle = \gamma\lambda T_N\delta(t - t')\delta(\mathbf{r} - \mathbf{r}') \qquad (S4)$$

where $T_N = T/M_0\mu_{Co}$ is the dimensionless "numerical temperature', T is temperature and μ_{Co} is the magnetic moment of a single Co atom. We simulate the stochastic equation (eq4) with the temperature gradually decreasing from the value $T_N = 3K_R$ to zero. The resulting "pseudo-ground" states are shown in **Fig. S7** for different parameters. It shows the crossover from magnetic domains and domain walls to CFG. They are used as the initial states for calculations of minor loops.

Although the general picture of zero-magnetization states (domains or spin glass) is robust against modification of the cooling procedure, the typical sizes of domains or of the spin-glass features depend on the time of the numerical cooling τ_{c0}. In Fig. S8 we show how the calculated state depends on this parameter. The value $\gamma\lambda\tau_{c0} = 10^3$ is used for the calculation of the minor loops.

To calculate the minor loops the adiabatic modification of field **H** starts from the demagnetized state. The magnetic field then is changed from zero to some value H_{max}. Three loops between H_{max} and $-H_{max}$ are made to saturate the magnetization dependence on the magnetic field and then the minor loop is recorded. The result is averaged over 500 disordered numerical samples. Then the minor loop is centered and coercive field H_C^* and remnant magnetization M_R^* are recorded. This procedure is shown in **Fig. S9**. Eqs. (S3) and (S4) allow to calculate the xy-magnetization properties. However, these equations do not allow magnetization to have z-component. Therefore, a vortex in this model are just a singularity in 2D energy density. To study the vortex structure, we apply the vector form of LLG equations.

$$\frac{\mathrm{d}\mathbf{M}}{\mathrm{d}t} = -\gamma[\mathbf{M} \times \tilde{\mathbf{H}}] - \frac{\gamma\lambda}{M_0}\left[\mathbf{M} \times [\mathbf{M} \times \tilde{\mathbf{H}}]\right]. \qquad (S5)$$

Here the Langevin forces are included in the effective magnetic field $\tilde{\mathbf{H}} = \partial\mathcal{F}/\partial\mathbf{M} + \mathbf{H}_\xi$, where the cartesian components $H_\xi^{(\alpha)}$of $\mathbf{H}_\xi$ have the following correlation function

$$\langle H_\xi^{(\alpha)}(\mathbf{r}, t)H_\xi^{(\beta)}(\mathbf{r}', t')\rangle = \frac{2T}{\gamma\lambda M_0}\delta_{\alpha\beta}\delta(t - t')\delta(\mathbf{r} - \mathbf{r}'). \qquad (S6)$$

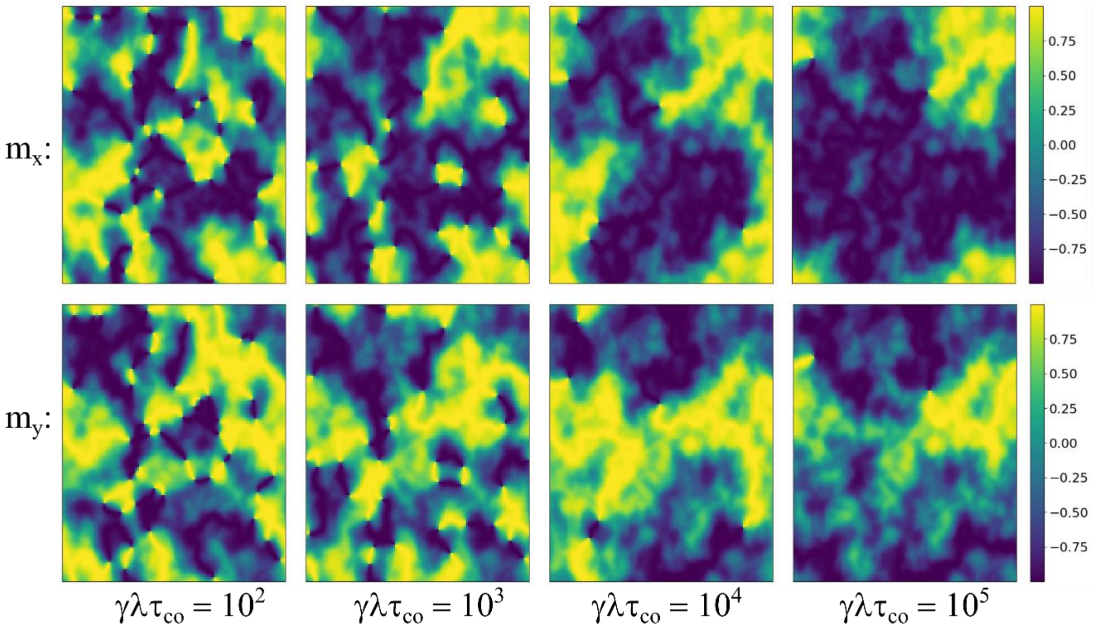

Fig. S8: dependence of the demagnetized state on the time of numerical cooling. All the states are calculated for $r_c = 2\Delta$ and $K_U = 0.02K_R$.

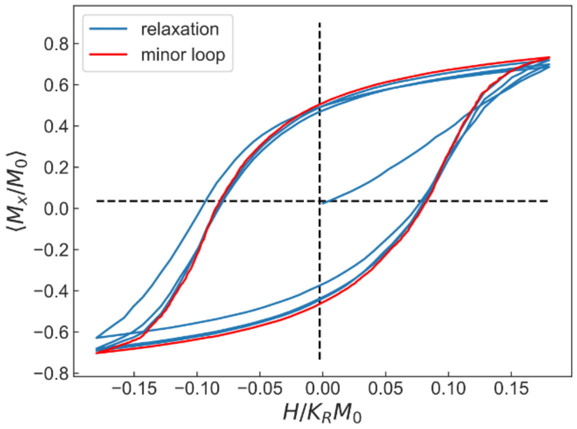

Fig. S9 Minor loop calculation.

To verify the validity of our model we have quantitatively compared the hysteresis loops at 150 K for Co/Al and Co/C_{60} with the micromagnetic model loops. Such comparison is reported in **Fig. S10**. For the experimental values of both samples we have rescaled the applied field by the anisotropy field 75 mT, estimated by looking at the closure field of the Co/C_{60} loop (i.e. the field for which the forward and backward branches are splitting). As can be seen from **Fig. S10**, The Co/C_{60} hysteresis loops is reasonably well reproduced by a K_R/K_U ratio of 50 and a correlation radius r_c=4Δ. Noteworthily, the Co/Al hysteresis loop is reproduced by the theoretical loops considering r_c=0.2Δ, that is a correlation radius that is 5 times less than the re-normalized magnetic length.

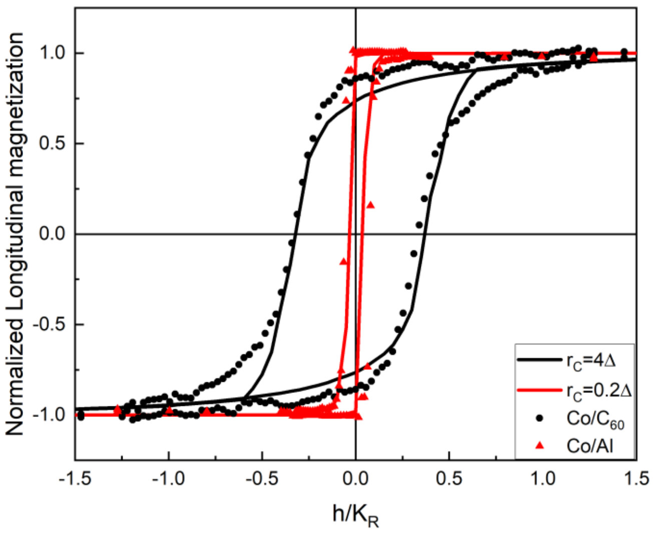

Fig. S10 Comparison between experimental hysteresis loops for Co/C_{60} (black points) and Co/Al (red points) and micromagnetic simulations considering an anisotropy ratio K_U/K_R=0.02 and magnetic length ratios r_c/Δ=4 (black line) and r_c/Δ=0.2. Experimental data measured at T=150K. Applied field is rescaled for both Co/Al and Co/C_{60} by the closure field of the latter.

S. V MAGNETIC FORCE MICROSCOPY CHARACTERIZATION

MFM measurements were taken at RT in an applied field of +2 and -2 mT, applied after a full magnetization reversal cycle with an opposite field of -60 mT and + 60 mT, respectively. **Fig. S11** shows both the topographic and the magnetic signal map on the Co(5nm)/C_{60}(25nm), highlighting how the magnetic signal is not affected by any morphological defect. For completeness we also report the enlarged area from which the data reported in **Fig.5 e** of the main text are taken. We also add 5 other profiles along 5 different vortexes to highlight the magnetic signal depth. The MFM magnetic signal image taken at -2mT is reported in **Fig. S12**, where peaks of opposite magnetic contrasts (w.r.t. the +2 mT case) but equivalent lateral dimensions are visible. For a better visualization we selected 4 of them and plotted their height profiles.

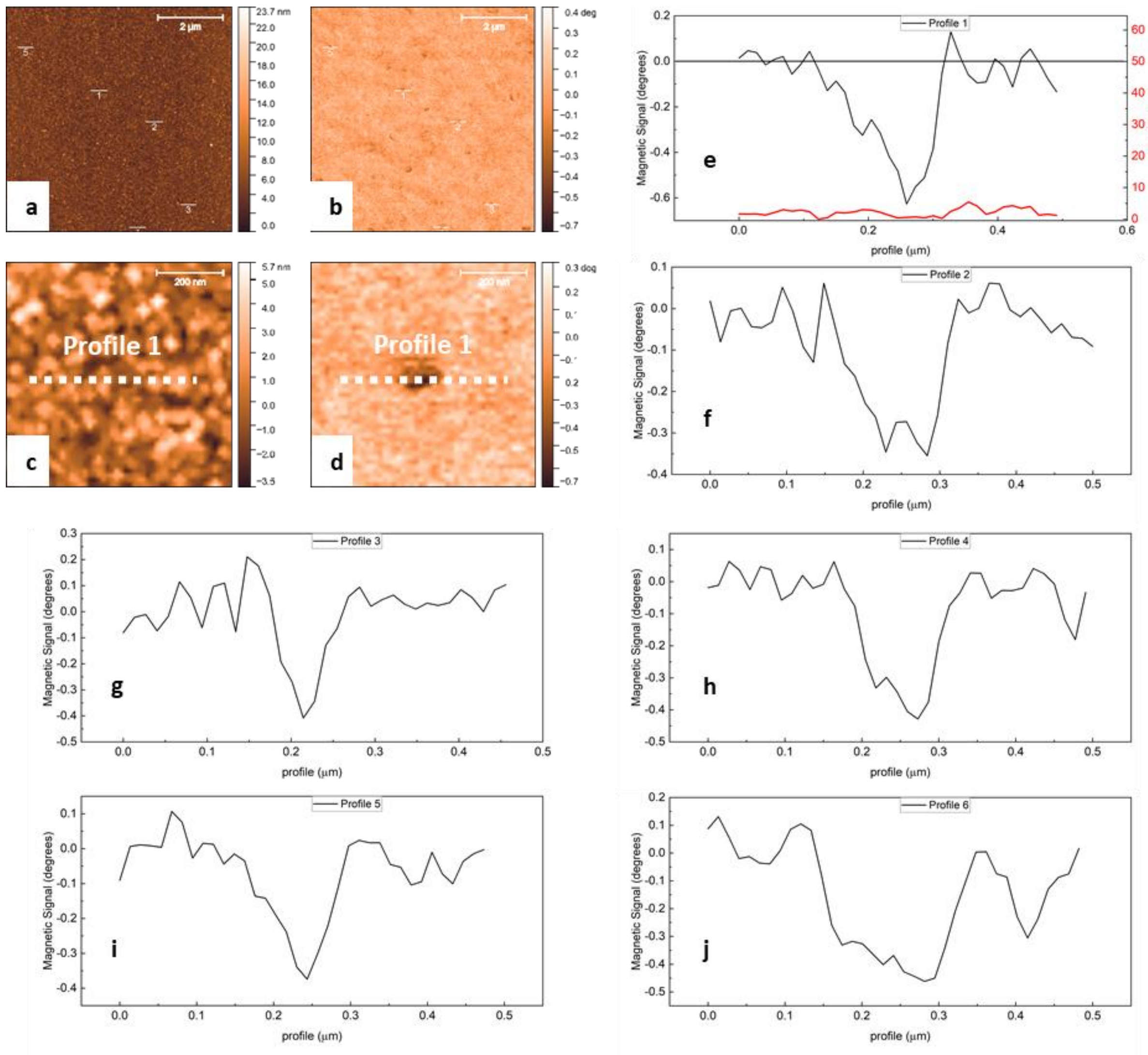

Fig. S11 a Topographic and **b** Magnetic signal maps of the Co/C_{60} sample. The area around Profile 1 (the one reported in **Fig.5 e** of the main text) is reported in **c** and **d** respectively. **e-j** report the 6 profiles of the magnetic signal taken along different magnetic vortexes. **e** also reports the corresponding topography profile with y-axis adjusted to highlight the 50 nm height (w.r.t. the surface) at which the Magnetic signal was taken.

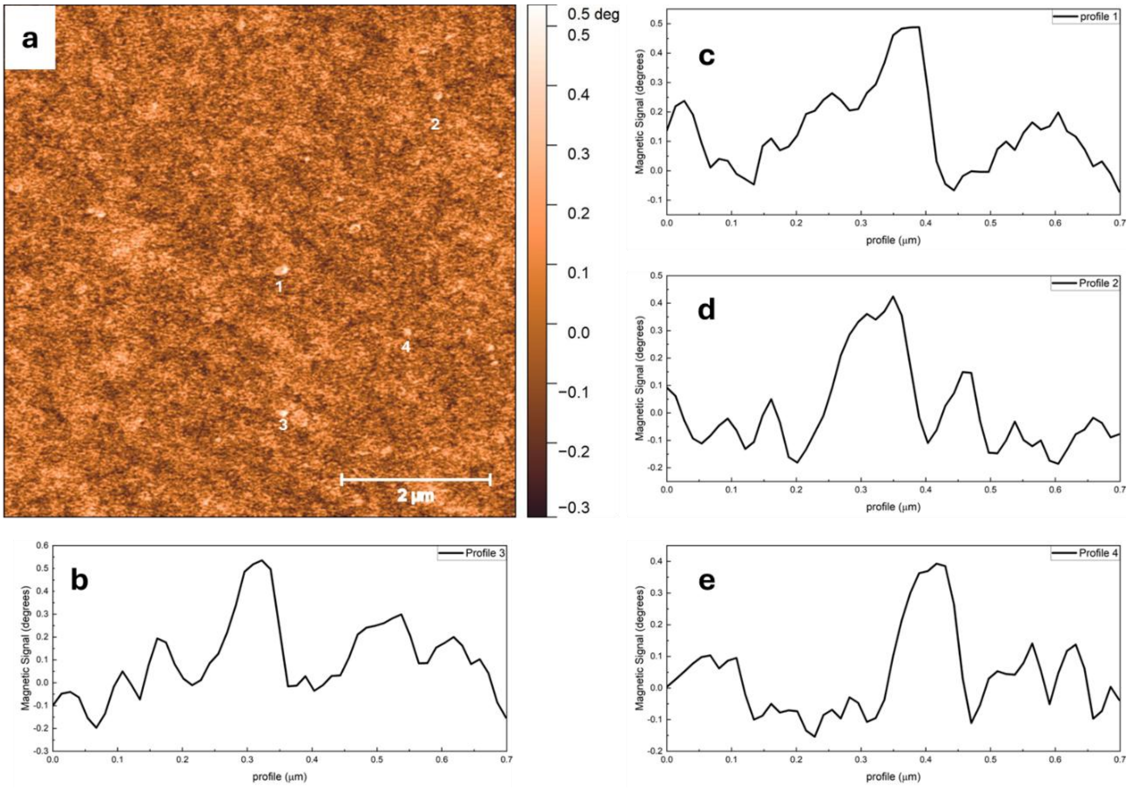

Fig. S12 a, Magnetic signal maps of the Co/C_{60} sample obtained with an applied field of -2 mT (opposite to the one in **Fig. S11**), along with 4 height profiles (**b-e**) each of which include a vortex-like defect (located above the white numbers in the image **a**).

S. VI RT and raw T=150 K hysteresis loops

In **Fig. S12 a** we report the hysteresis loops of Co(5nm) samples at RT, showing an increase in coercivity accompanied by enlarged loop area. For completeness, we report also the non-symmetrized version of the loops shown in **Fig. 1a** of the main article in **Fig. S13 b**, along with the other Co/C_{60} and Co/Gaq_3 loops used to extract the coercivities reported in **Fig. 5** of the main text. Of particular interest are the Co/Gaq_3 loops, showing (for the loop reported in **Fig. 1a**) no clear double jump but a "peak" at μ_0H=0 mT that is attributed to an artifact of the measurement. In fact, by inspecting the raw hysteresis loops (**Fig. S14**) used for the averaged Co/Gaq_3 loop at 150 reported in **Fig. 1a** of the main article, a spurious oscillating signal is present in the voltage measured by the MOKE photodetector. Its presence affected the average loop resulting in an artificial kink around H=0. Note moreover that the other loop obtained for a different Co/Gaq_3 sample does not show any double phase behaviour of the magnetization reversal

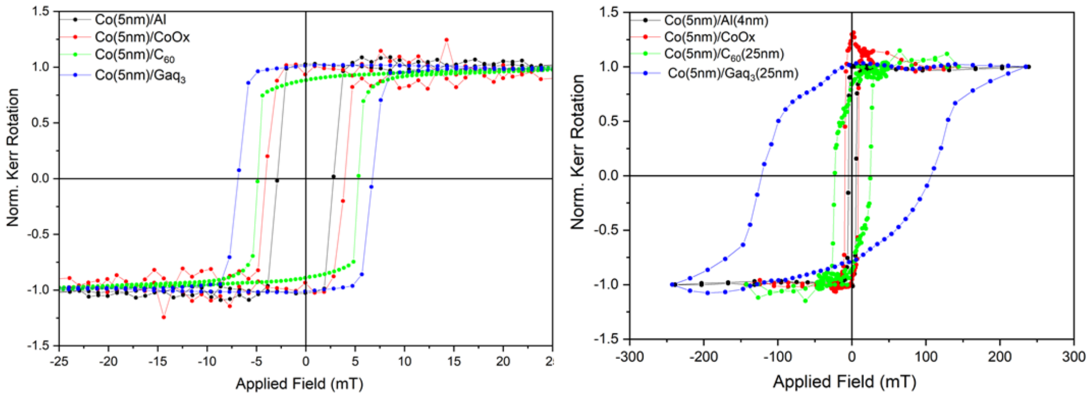

Fig. S13 a RT hysteresis loops of Co(5nm) samples. **b** non-symmetrized, normalized hysteresis loops of Co(5nm) samples measured at T=150K (same data shown in Fig. 1).

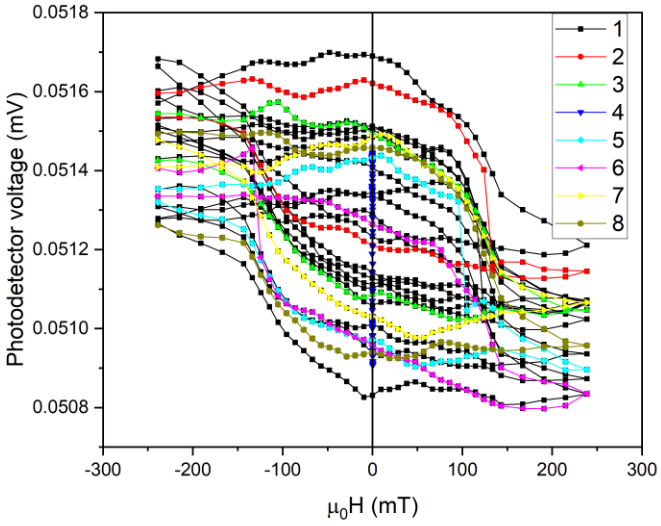

Fig S14 raw data of the Photodetector output voltage vs applied magnetic field for each averaged loops in the final Co/Gaq3 loop measured at 150 K. A clear oscillating spurious signal is present, affecting the true shape of the hysteresis loop of the sample. Its inclusion in the average process gave rise to the kink appearing in the return branch (H+ to H-)of the hysteresis loop reported in **Fig. S12 b**.

S. VII GRAIN SIZE and RT, 100 K coercivity for 3,5 and 7 nm thick Co layers

We report in Table S1 the valiues of the mean grain size for 3, 5 an 7 nm thick Co layers. They were extracted by the procedure reported in Rasigni *et al.*[3], by fitting the 1D fast-axis autocorrelation function of 1 μm Co surfaces, obtained by ex-situ AFM at RT. The estimated grain sizes are compatible, for different thicknesses, within the error bars. We also report in Table S1 the values of the coercivity of Co/C_{60} samples at RT and 80 K.

**Co thickness t (nm)**	3	5	7
**Mean grain size (nm)**	9 ± 1	11 ± 1	11 ± 1
**RT Co(t)/C_{60} $\mu_0 H_C$ (mT)**	6.5 ± 0.4	4.7 ± 0.3	3.2 ± 0.4
**RT Co(t)/Al $\mu_0 H_C$ (mT)**	4.6 ± 0.4	3.6 ± 0.3	3.1± 0.4
**80K Co(t)/C_{60} $\mu_0 H_C$ (mT)**	227 ± 25	84 ± 5	9.6 ± 0.4
**80K Co(t)/Al $\mu_0 H_C$ (mT)**	9.5 ± 0.5	4.8 ± 0.4	5.0 ± 0.3

Table S1: Mean Grain size of Co layer and coercivities of Co/C_{60} samples as a function of the Co thickness. Coercivity errors: magnetic field sweep stepsize. Mean grain size error bars obtained by nonlinear fitting.

References (supplementary)

1. Harris, R., Plischke, M. & Zuckermann, M. J. New Model for Amorphous Magnetism. *Phys. Rev. Lett.* **31**, 160–162 (1973).

2. Dimitrov, D. A. & Wysin, G. M. Effects of surface anisotropy on hysteresis in fine magnetic particles. *Phys. Rev. B* **50**, 3077–3084 (1994).

3. Rasigni, G. *et al.* STATISTICAL PARAMETERS FOR RANDOM AND PSEUDORANDOM ROUGH SURFACES. *J. Opt. Soc. Am. A-Opt. Image Sci. Vis.* **5**, 99–103 (1988).
